# Photographic intervention effect on positive and negative affects during COVID-19: Mediating role of future self-continuity

**DOI:** 10.3389/fpsyg.2022.1085518

**Published:** 2023-01-04

**Authors:** Feng Zhang, Yu Pi, Xiaobao Li

**Affiliations:** Department of Psychology and Institute of Psychology and Behavior, Henan University, Kaifeng, China

**Keywords:** photographic intervention, meaning in life, positive and negative affects, future self-continuity, mediating effect

## Abstract

Meaning in Life (MIL) is a protective factor that buffers the impact of COVID-19 epidemic on emotions. Our study aimed to explore whether photographic intervention based on MIL could increase Positive Affect (PA) and mitigate Negative Affect (NA), and whether Future Self-Continuity (FSC) functioned as a mediator between them. In this study, 90 college students were randomly divided into an intervention group or a control group. Participants in the intervention group were asked to take a photo and describe it every 2 days lasting 2 weeks. All the participants in the two groups were measured by the Meaning in Life Questionnaire, Positive and Negative Affect Scale, and Future Self-Continuity Scale before and after the intervention. The results showed that: (1) Before the intervention, there were no significant differences in baseline levels of MIL, PA and NA, and FSC between intervention and control groups. (2) In the intervention group, compared to pre-test, the scores of MIL, PA, and FSC of post-test increased significantly, and the score of NA of post-test decreased significantly. (3) After the intervention, the scores of MIL, PA, and FSC in the intervention group were significantly higher than those in the control group; NA score in the intervention group was significantly lower than that in the control group. (4) In terms of the difference score (post-test minus pre-test), FSC was a mediator between MIL and PA. Our study demonstrated that photographic intervention could effectively improve college students’ MIL, PA, and FSC while mitigating NA. Moreover, MIL could significantly enhance PA by the mediating role of FSC.

## 1. Introduction

The COVID-19 outbreak had a significant negative impact on people’s mental health (Gan et al., 2022). Meaning in life (MIL) was a protective factor that buffered the impact of the epidemic on general health (e.g., state anxiety and COVID-19 stress) and life satisfaction (De Jong et al., 2020; Trzebiński et al., 2020). MIL was defined as “the sense made of, and significance felt regarding, the nature of one’s being and existence” (Steger et al., 2006) and was one of the most fundamental variables that contributed to a pleasant and positive mood (Hone et al., 2014; King and Hicks, 2021). Frankl (1985) proposed that searching for the MIL was the original driving force of human life. Based on Seligman’s (2002) theory of happiness, a meaningful life was the ultimate way to achieve genuine happiness and life satisfaction. Numerous studies have supported beneficial effects of having more MIL on individuals’ positive and negative emotions (Updegraff et al., 2008; Miao et al., 2017; Hooker et al., 2018). For instance, studies showed that having more MIL was closely related to an optimistic attitude toward life (Steger and Kashdan, 2007), more positive social interactions (Steger and Shin, 2010), and increased life satisfaction (Bonebright et al., 2000). On the contrary, having less MIL could lead to one’s negative psychological outcomes, such as depression (Steger et al., 2009), anxiety (Steger and Kashdan, 2007), and even suicidal tendencies (Steger et al., 2008). Therefore, enhancing MIL might be an effective means to improve positive affect (PA) and reduce negative affect (NA).

Earlier studies used self-report measures to assess MIL at a certain time point (Peterson et al., 2005; Steger and Shin, 2010). Given that the previous method was hard to deeply get one’s overall sense of meaning because of a lack of dynamic evaluation of experiencing and living in life, Steger et al. (2013) developed a visual research method of photographic intervention to assess one’s MIL and tested its effectiveness on positive and negative affects. The photographic intervention was constructed from the perspective of improving individuals’ cognition and emotional evaluation of life’s purpose and sense of value (Steger et al., 2013, 2014). In the 1-week intervention, participants were asked to take photos that made their lives meaningful, and then described the significance of all these photos at the end of the week, and the results indicated that photographic intervention promoted individuals’ MIL that was significantly related to increased PA, increased life satisfaction, and reduced NA (Steger et al., 2014); however, asking participants to describe all the pictures a week later might cause memory burden and biases (Steger et al., 2014; Miao and Gan, 2019). Even more, the lack of a control group in Steger et al. (2014) made it impossible to exclude other factors’ potential effects. Therefore, the present study was designed to retest the effect of photographic intervention on positive and negative affects during COVID-19 by adding a control group and asking participants to write a description of the photo in time.

Although current evidence has proven the positive effect of photographic intervention on individuals’ emotions, the underlying mediation mechanism remains to be further explored. Our study speculated that future self-continuity (FSC) might play a mediating role between MIL and PA/NA. FSC meant the connection between one’s present self and future self, and it was a key component of a global self-continuity or a stable identity (Van Gelder et al., 2013). FSC was closely related to behavioral outcomes, such as more financial saving behaviors, greater academic achievement, less delinquent behaviors, and healthy exercise behaviors (Ersner-Hershfield et al., 2009; Van Gelder et al., 2013; Rutchick et al., 2018). This might be because individuals with higher FSC were more likely to see the future as hopeful and attainable, and to have the willingness and ability to engage in adaptive behaviors in the present for achieving an obtainable future self (Sokol and Serper, 2019). First, Baumeister et al. (2013) stated that one of the most critical functions of MIL was temporal integration of the past, present, and future. Research indicated that seeking and discovering a meaningful life was linked with the ability to maintain and enhance the self-continuity between the present and the future (King and Hicks, 2021). Second, increased FSC contributed individuals to constructively processing negative life events, such as adopting a broader temporal perspective to reduce self-esteem declines (Wink and Schiff, 2002; Sokol and Serper, 2019). The research results indicated that individuals with more overlapping between present self and future self were more prone to positively view their future (Zhang and Aggarwal, 2015) which could directly bring positive emotions (Bryant, 2003), high levels of FSC improved PA and life satisfaction (Sokol and Serper, 2019), while low levels of FSC were strongly associated with NA and suicidal ideation (Sokol and Eisenheim, 2016; Sokol and Serper, 2017, 2019). Third, Terror Management Theory (TMT), based on existential psychodynamic tradition, indicated that one’s death was always potentially imminent and inevitably in conflict with motivational systems geared toward continued life, thus triggering personal anxiety (Greenberg et al., 2008). During COVID-19 epidemic, individuals might experience psychologically the fear related to death. Frankl (1985); Becker (1973) stated that over time, the perception of self-identity and continuity of meaning psychologically protected individuals from the awareness of death threats. Studies results showed that mortality salience led high self-concept structure seekers to prefer causal consistency of recent experiences and meaningful connections between past events and the current self (Landau et al., 2009). Therefore, the continuous search for MIL can maintain self-identity in temporal self, inspiring one’s positive expectations for the future life and alleviating negative emotions. Based on the above research and theory, MIL could improve emotion by the mediating role of FSC.

The present study aimed to explore the effect of photographic intervention based on MIL on college students’ positive and negative affects, and FSC, and whether FSC played a mediating role between MIL and emotions. The photographic intervention, as a MIL-based approach, could draw attention to current life experiences and continuous recording of meaningful life events. Through the constant searching for meaningful information about life, people developed a continuous and uninterrupted identity about their lives, that was, they drew a close connection between their present and future lives (higher FSC), which improved their emotions. We proposed three hypotheses: (a) The MIL-based photographic intervention would enhance participants’ MIL; (b) The intervention would increase the levels of PA and FSC, and mitigate the level of NA; and (c) FSC would mediate the relationship between MIL and PA/NA. In our study, a control group was added, and the participants in the intervention group were asked to complete a 14-day photographic intervention, and they were required to upload photos and describe the content of the photos to reduce the memory burden and memory bias.

## 2. Materials and methods

### 2.1. Participants

A total of 36 participants were required by calculating the sample size using a G^*^power analysis (version 3.1.9, *f* = 0.25, *α* = 0.05, 1-*β* = 0.95). Ninety college students as volunteer participants were recruited during COVID-19 epidemic, and randomly assigned to an intervention group or a control group. The age of 45 participants in the intervention group ranged from 18 ~ 21 years old (36 females; *M* = 19.31; *SD* = 0.70), and the age of 45 participants in the control group ranged from 18 ~ 20 years old (33 females; *M* = 19.16; *SD* = 0.74). There were no significant differences in gender (*t* = 0.74, *p* = 0.460) and age (*t* = 1.03, *p* = 0.308) between the two groups. Informed consent was obtained from each student prior to the study. The study was conducted in accordance with the ethical standards of the institutional research committee.

### 2.2. Measures

#### 2.2.1. Meaning in life questionnaire

MIL was assessed using a Chinese version of Meaning in Life Questionnaire (MLQ; Liu and Gan, 2010). This questionnaire comprised 9 items with two dimensions: The presence of meaning and the search for meaning. All items were rated on a 7-point Likert scale (1 = “absolutely untrue” or “very slightly” to 7 = “absolutely true” or “extremely”). At pre-test, the *α* coefficient for the questionnaire was 0.78. At post-test, the *α* coefficient was 0.91.

#### 2.2.2. Future self-continuity scale

Future Self-Continuity Scale (FSCS) adapted by Ersner-Hershfield et al. (2009) was used. This visual scale contained seven pair circles labeled “current self” and “future self,” ranging from complete separation (1 = least similar) to almost complete overlap (7 = most similar). Participants were asked to choose one of seven pairs of circles to represent the proximity of their present self and future self. The more the overlap between two circles, the higher the FSC level. This instrument was the most commonly used and currently the most validated FSC measure with sufficient test–retest reliability, and it could significantly predict performance on structures associated with FSC (Ersner-Hershfield et al., 2009).

#### 2.2.3. Positive and negative affect scale

The Chinese version of the Positive and Negative Affect Scale (PANAS) consisted of two dimensions: Positive affect (such as interested and enthusiastic) and negative affect (such as guilty and irritable), including 20 items (Huang et al., 2003). All the items were rated on a 5-point Likert scale (from 1 = “very slightly” to 5 = “very much”). At pre-test, the *α* coefficients for positive affect and negative affect were 0.80 and 0.83, respectively. At post-test, the *α* coefficients were 0.78 and 0.87, respectively.

### 2.3. Procedures

In this study, offline questionnaire surveys using MLQ, FSCS, and PANAS were conducted for all participants before the intervention (pre-test) and after the intervention (post-test).

In the intervention group, a 2-week intervention was implemented. They were asked to take photos and answer questions online every 2 days. Specifically, the participants were instructed to take photos of “things that make you feel meaningful in your life” with their smartphones. After each photo shoot, they were asked to upload the photos and online answer the following questions based on photo contents: “(1) What does this photo represent? (2) Why does it make you feel that your life is meaningful?” through a dedicated secure website. At 8:00 AM, they were reminded to take photos, and at 9:00 PM they were reminded to upload the photos they had taken to a designated website and answer corresponding questions.

In the control group, participants did not receive the intervention for 14 days. To exclude the expectation effect of the control group, we did not inform participants of the purpose of completing the questionnaires at pre-test and post-test.

### 2.4. Data analyzes

All the analyzes were performed using SPSS 23.0 software. An independent sample *t*-test was conducted to compare the differences in baseline scores between the two groups for baseline comparison. One-way multivariate analysis of variance (MANOVA) was used to examine the pre-test to post-test change scores. Analyzes of covariance (ANCOVA) were conducted to examine the increase in PA and decrease in NA after the intervention phase. Correlation analysis was performed for all variables, and Hayes (2018) Process Macro (Model 4) was used to conduct mediation analysis. The bootstrapping method was applied to test the mediating effect. The significance level of all variables was set as *ɑ* = 0.05.

## 3. Results

### 3.1. Baseline comparison

The results of the independent samples *t*-test showed that there was no significant difference in the baseline scores of MIL, FSC, and PA/NA between intervention and control groups (see Table 1), indicating that the randomization procedure was effective and there was no significant difference at baseline level between the two groups.

**Table 1:** Baseline scores between intervention and control groups (*M* ± SD).

	Intervention group	Control group	*t*	*p*	Cohen’s *d*
MIL	37.42 ± 7.02	38.91 ± 6.21	−1.07	0.290	−0.22
FSC	3.56 ± 1.32	3.98 ± 1.22	−1.58	0.119	−0.33
PA	27.60 ± 4.90	29.04 ± 3.91	−1.55	0.126	−0.32
NA	28.20 ± 5.73	27.22 ± 5.27	0.84	0.401	0.18

### 3.2. The effect of photographic intervention

We examined difference scores (post-test minus pre-test) to determine whether the intervention enhanced MIL, FSC, and PA, and whether the intervention reduced NA. Means and standard deviations of pre-test and post-test scores between intervention and control groups were presented in Table 2. MANOVA for difference scores on the three questionnaires of MLQ, FSCS, and PANAS revealed significant multivariate main effect of group (control group, intervention group), Wilks’ lambda = 0.41, *F*_(4,85)_ = 30.94, *p* < 0.001, *η_p_^2^ =* 0.593. An examination of the univariate tests demonstrated significant differences: MIL, *F*_(1,88)_ = 45.02, *p* < 0.001, *η_p_^2^ =* 0.338; FSC, *F*_(1,88)_ = 26.74, *p* < 0.001, *η_p_^2^ =* 0.233; PA, *F*_(1,88)_ = 71.80, *p* < 0.001, *η_p_^2^ =* 0.449; and NA, *F*_(1,88)_ = 25.23, *p* < 0.001, *η_p_^2^ =* 0.223. As predicted, compared to the control group, the intervention group showed significant increases in MIL, FSC, and PA, as well as significant decreases in NA.

**Table 2:** Pre-test and post-test scores between intervention and control groups (*M* ± SD).

Variables	Intervention group		Control group		*t*	Cohen’s *d*
	Pre-test	Post-test	Pre-test	Post-test		
MIL	37.42 ± 7.02	45.96 ± 5.10	38.91 ± 6.21	36.38 ± 7.29	−6.71^***^	−1.42
FSC	3.56 ± 1.32	5.29 ± 1.12	3.98 ± 1.22	3.93 ± 1.30	−5.17^***^	−1.09
PA	27.60 ± 4.90	35.44 ± 3.41	29.04 ± 3.91	29.51 ± 3.38	−8.47^***^	−1.79
NA	28.20 ± 5.73	22.60 ± 5.29	27.22 ± 5.27	28.82 ± 4.73	5.02^***^	1.06

***p < 0.001.

The results of a one-way ANCOVA for PA, with groups (control group, intervention group) as the independent variable and PA at pre-test as the covariate, revealed significant differences between two conditions, *F*_(1,88)_ = 100.33, *p* < 0.001, *η_p_^2^ =* 0.536. Another one-way ANCOVA for NA, with groups (control group, intervention group) as the independent variable and NA at pre-test as the covariate, indicated significant differences between two groups, *F*_(1,88)_ = 36.60, *p* < 0.001, *η_p_^2^ =* 0.296. Tukey HSD *post hoc* tests indicated that the score of PA in intervention group was significantly higher than that in control group, and the score of NA in intervention group was significantly lower than that in control group.

### 3.3. The mediating effect of FSC

Before conducting the mediation analysis, a correlation analysis was performed to examine the relationship among the variables (see Table 3). MIL was significantly positively related to FSC and PA, respectively. FSC was significantly positively related to PA. NA was significantly negatively related to MIL and PA, respectively.

**Table 3:** Correlations among the variables at pre-test and post-test.

Variables	1	2	3	4	5	6	7	8
1.pre_MIL	1							
2.pre_FSC	0.29^**^	1						
3.pre_PA	0.41^**^	0.23^*^	1					
4.pre_NA	−0.15	−0.12	−0.13	1				
5.post_MIL	0.14	−0.09	0.06	0.13	1			
6.post_FSC	0.06	0.13	−0.25^*^	−0.15	0.33^**^	1		
7.post_PA	0.11	−0.21^*^	0.24^*^	−0.07	0.56^**^	0.54^**^	1	
8.post_NA	0.10	0.02	0.12	0.09	−0.45^**^	−0.14	−0.22^*^	1

Alpha coefficients are presented in the diagonal. ^**^p < 0.01; ^*^p < 0.05.

PROCESS Model 4 was used to test the mediating role of FSC. According to Liu et al. (2021), our study used difference scores (post-test minus pre-test) for mediation analysis. Before entering the model, difference scores of MIL, FSC, PA, and NA were normalized to obtain standardized parameter estimates. In addition, age and gender were entered into the model as covariates. Gender was created as a dummy variable (female = 1, male = 0). The results (see Table 4) showed that the direct effects of MIL on PA/NA and the indirect effect of MIL on PA were significant, however, the indirect effect of MIL on NA did not reach significance.

**Table 4:** Results of mediation analysis.

Dependent variables	Predictors	*β*	*SE*	*t*
FSC	MIL (a)	0.31	0.10	3.08^**^
	Gender	0.45	0.23	1.90
	Age	−0.25	0.14	−1.76
PA	MIL (c’)	0.34	0.08	4.26^***^
	FSC (b)	0.56	0.08	6.81^***^
	Gender	−0.14	0.18	−0.77
	Age	0.08	0.11	0.73
	Indirect effect (ab)	0.17	0.08	[0.05, 0.36]
NA	MIL (c’)	−0.52	0.10	−5.23^***^
	FSC (b)	0.13	0.10	1.27
	Gender	−0.13	0.23	−0.59
	Age	−0.12	0.13	−0.86
	Indirect effect (ab)	0.04	0.03	[−0.01, 0.12]

^**^p < 0.01, ^***^p < 0.001.

**Figure 1:**
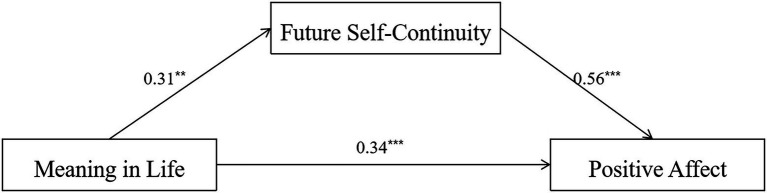
Mediational pathways of MIL on PA. ^**^p < 0.01, ^***^p < 0.001.

The model fit was significant when the dependent variable was PA, *R^2^* = 0.26, *F*_(3,86)_ = 10.05, *p* < 0.001. The total effect of MIL on PA was significant, *β* = 0.51, *SE* = 0.09, 95% CI = [0.32, 0.69], and the direct effect was also significant, *β* = 0.34, *SE* = 0.08, 95% CI = [0.18, 0.50]. As expected, we found a significant indirect effect of MIL on PA *via* FSC, ab = 0.17, *SE* = 0.08, 95%CI [0.05, 0.36], indicating that FSC served as a mediator in this relationship, and FSC played a partial mediating role between MIL and PA (see Figure 1).

**Figure 2:**
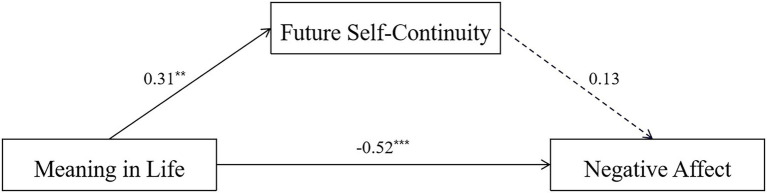
Mediational pathways of MIL on NA. ^**^p < 0.01, ^***^p < 0.001.

The total effect of MIL on NA was significant, *β* = −0.48, *SE* = 0.09, 95% CI = [−0.66, −0.29], and the direct effect was also significant, *β* = −0.52, *SE* = 0.10, 95% CI = [−0.71, −0.32], however, the indirect effect of MIL on NA through FSC did not reach significance, ab = 0.04, *SE* = 0.03, 95% CI [−0.01, 0.12]. Therefore, FSC in MIL and NA did not seem to fit the mediation model, suggesting that FSC did not play a mediating role between MIL and NA (see Figure 2).

## 4. Discussion

In order to overcome the shortcomings of previous study (Steger et al., 2014), our study added a control group and increased the frequency of photography. Through a 2-week intervention, we verified that photographic intervention based on MIL promoted PA and reduced NA, and that FSC played a mediating role between MIL and PA. Consistent with previous findings (Steger et al., 2014; Miao and Gan, 2019), our study demonstrated that photographic intervention was an effective way to improve emotions during COVID-19.

FSC was explored as an acting mechanism between photographic intervention and PA in the present study. First, we found that photographic intervention increased individuals’ FSC, demonstrating the conducive effect of MIL on FSC. This finding was in line with previous studies (Rubin et al., 2016, 2019) positing that individuals with a high sense of current life experiences tended to exhibit a psychological state that was more closely connected with the future, and that individuals with a low sense of life experience could only isolate the current meaningful life and cannot connect well with future self (Rubin et al., 2016). The photographic intervention guided participants to express the meaning of the photographs coherently and to gain a deeper understanding of their own daily lives, and the coherent expression gave meaning to life transformation and achieving self-continuity (Hong et al., 2021; King and Hicks, 2021). Thus, over time, the experience of MIL might gradually deepen one’s perception of the connection of present self and future self. Second, the sense of self-continuity might play a crucial role in guiding emotional responses (Sadeh and Karniol, 2012), and increasing self-continuity could enhance subjective well-being by shifting to a broader temporal perspective (Sokol and Serper, 2019). Therefore, photographic intervention used a coherent expression of a meaningful life to achieve self-identity in time, generate psychological pleasure, and improve their overall satisfaction with life. This meant that individuals with a higher awareness of MIL could establish a closer connection with the future, thereby enhancing the positive affect on their overall life. As expected, our results confirmed the mediating role of FSC between MIL and PA.

However, we found that FSC did not play a mediating role in this relationship between MIL and NA. Taking photos could be a clear record of a meaningful life, and expressive writing helped to transform abstract MIL (generated from photos) into a narrative and emotional (usually positive) language (Steger et al., 2014; Miao and Gan, 2019). Thus, photographic intervention enhanced primarily positive emotion in our study. According to the “Undoing Hypothesis,” based on the Broaden-and-Build theory (Fredrickson et al., 2000; Fredrickson, 2001), experiencing positive affects can “eliminate” or “undo” the negative effects of experiencing negative affects (Pezirkianidis et al., 2016). As a result, the mediating effect of FSC between MIL and NA did not reach significance in the present study. Future study should explore the photographic intervention aimed to reducing NA, and reexamine the mediating role of FSC.

Our study had several implications. Photographic intervention focused on increasing MIL contributed to an individual’s emotions and well-being. During COVID-19, MIL should be constructed in a variety of ways to increase PA and reduce NA. In addition, improving an individual’s MIL through photographic intervention led to an increasing of FSC, in turn, enjoying a whole and positive life. Previous findings suggested that interventions aimed to increase self-continuity could promote an individual’s self-concept and subjective well-being (Sokol and Serper, 2017, 2019). Attention should be paid to improving individuals’ FSC to promote emotional regulation during COVID-19.

There were some limitations in our study. First, the participants were from a university. One should be cautious about generalizing our findings to other groups. Second, the sources of college students’ MIL might be diverse. In future research, the intervention effects of different types of MIL should be investigated to develop more effective intervention programs. Third, this study did not provide dynamic measurements of intervention effects. In future studies, researchers could adopt an experience sampling method to collect immediate data to examine the effectiveness of photographic intervention.

## Data availability statement

The original contributions presented in the study are included in the article/Supplementary material, further inquiries can be directed to the corresponding author.

## Ethics statement

The studies involving human participants were reviewed and approved by the Ethics Committee of Henan Province Key Laboratory of Psychology and Behavior. The participants provided their written informed consent to participate in this study.

## Author contributions

FZ and YP contributed to conception and design of the study. YP and XL organized the database, performed the statistical analysis, and wrote the first draft of the manuscript. All authors contributed to manuscript revision, read, and approved the submitted version.

## Funding

This project was supported by the National Social Science Fund of China (No. 18BSH112).

## Conflict of interest

The authors declare that the research was conducted in the absence of any commercial or financial relationships that could be construed as a potential conflict of interest.

## Publisher’s note

All claims expressed in this article are solely those of the authors and do not necessarily represent those of their affiliated organizations, or those of the publisher, the editors and the reviewers. Any product that may be evaluated in this article, or claim that may be made by its manufacturer, is not guaranteed or endorsed by the publisher.
